# Supplementary material for: A Critical View of the Application of the APEX Software (Aqueous Photochemistry of Environmentally-Occurring Xenobiotics) to Predict Photoreaction Kinetics in Surface Freshwaters
Source: Molecules. 2019 Dec 18;25(1):9. doi: 10.3390/molecules25010009 (PMC7017383; doi:10.3390/molecules25010009)
Supplement: Supplementary file 1 [file molecules-25-00009-s001.zip › 3.2.4_gcc-4.4.0.zip!/3.2.4_gcc-4.4.0/3.2.4_gcc-4.4.0/doc/octave/html/interpreter/Functions-of-Multiple-Variables.html]

Functions of Multiple Variables - Untitled


Next: Orthogonal Collocation,
Previous: Functions of One Variable,
Up: Numerical Integration


---

### 22.3 Functions of Multiple Variables

Octave does not have built-in functions for computing the integral of
functions of multiple variables directly. It is however possible to
compute the integral of a function of multiple variables using the
functions for one-dimensional integrals.

To illustrate how the integration can be performed, we will integrate
the function

```
     f(x, y) = sin(pi*x*y)*sqrt(x*y)
```

for x and y between 0 and 1.

The first approach creates a function that integrates f with
respect to x, and then integrates that function with respect to
y. Since `quad` is written in Fortran it cannot be called
recursively. This means that `quad` cannot integrate a function
that calls `quad`, and hence cannot be used to perform the double
integration. It is however possible with `quadl`, which is what
the following code does.

```
     function I = g(y)
       I = ones(1, length(y));
       for i = 1:length(y)
         f = @(x) sin(pi.*x.*y(i)).*sqrt(x.*y(i));
         I(i) = quadl(f, 0, 1);
       endfor
     endfunction
     
     I = quadl("g", 0, 1)
           => 0.30022
```

The above process can be simplified with the `dblquad` and
`triplequad` functions for integrals over two and three
variables. For example

```
     I =  dblquad (@(x, y) sin(pi.*x.*y).*sqrt(x.*y), 0, 1, 0, 1)
           => 0.30022
```

— Function File: **dblquad** (f, xa, xb, ya, yb, tol, quadf, ...)  
> Numerically evaluate a double integral. The function over with to
> integrate is defined by f, and the interval for the
> integration is defined by `[`xa`,` xb`,` ya`,` yb`]`. The function f must accept a vector x and a
> scalar y, and return a vector of the same length as x.
>
> If defined, tol defines the absolute tolerance to which to
> which to integrate each sub-integral.
>
> Additional arguments, are passed directly to f. To use the default
> value for tol one may pass an empty matrix.
>
>
>
> **See also:** triplequad, quad, quadv, quadl, quadgk, trapz.

— Function File: **triplequad** (f, xa, xb, ya, yb, za, zb, tol, quadf, ...)  
> Numerically evaluate a triple integral. The function over which to
> integrate is defined by f, and the interval for the
> integration is defined by `[`xa`,` xb`,` ya`,` yb`,` za`,` zb`]`. The function f must accept a
> vector x and a scalar y, and return a vector of the same
> length as x.
>
> If defined, tol defines the absolute tolerance to which to
> which to integrate each sub-integral.
>
> Additional arguments, are passed directly to f. To use the default
> value for tol one may pass an empty matrix.
>
>
>
> **See also:** dblquad, quad, quadv, quadl, quadgk, trapz.

The above mentioned approach works but is fairly slow, and that problem
increases exponentially with the dimensionality the problem. Another
possible solution is to use Orthogonal Collocation as described in the
previous section. The integral of a function f(x,y) for
x and y between 0 and 1 can be approximated using n
points by
the sum over `i=1:n` and `j=1:n` of `q(i)*q(j)*f(r(i),r(j))`,
where q and r is as returned by `colloc(n)`. The
generalization to more than two variables is straight forward. The
following code computes the studied integral using n=7 points.

```
     f = @(x,y) sin(pi*x*y').*sqrt(x*y');
     n = 7;
     [t, A, B, q] = colloc(n);
     I = q'*f(t,t)*q;
           => 0.30022
```

It should be noted that the number of points determines the quality
of the approximation. If the integration needs to be performed between
a and b instead of 0 and 1, a change of variables is needed.
